# Bacterial exopolysaccharides: biosynthesis pathways and engineering strategies

**DOI:** 10.3389/fmicb.2015.00496

**Published:** 2015-05-26

**Authors:** Jochen Schmid, Volker Sieber, Bernd Rehm

**Affiliations:** ^1^Chair of Chemistry of Biogenic Resources, Technische Universität MünchenStraubing, Germany; ^2^Institute of Fundamental Sciences, Massey UniversityPalmerston North, New Zealand; ^3^The MacDiarmid Institute for Advanced Materials and NanotechnologyPalmerston North, New Zealand

**Keywords:** bacterial exopolysaccharides, tailor-made exopolysaccharides, polysaccharide engineering, biosynthesis, gene clusters

## Abstract

Bacteria produce a wide range of exopolysaccharides which are synthesized via different biosynthesis pathways. The genes responsible for synthesis are often clustered within the genome of the respective production organism. A better understanding of the fundamental processes involved in exopolysaccharide biosynthesis and the regulation of these processes is critical toward genetic, metabolic and protein-engineering approaches to produce tailor-made polymers. These designer polymers will exhibit superior material properties targeting medical and industrial applications. Exploiting the natural design space for production of a variety of biopolymer will open up a range of new applications. Here, we summarize the key aspects of microbial exopolysaccharide biosynthesis and highlight the latest engineering approaches toward the production of tailor-made variants with the potential to be used as valuable renewable and high-performance products for medical and industrial applications.

## Introduction

Polysaccharides produced by microbes can be generally classified by their biological functions into intracellular storage polysaccharides (glycogen), capsular polysaccharides which are closely linked to the cell surface (e.g., K30 O-Antigen) and extracellular bacterial polysaccharides (for example, xanthan, sphingan, alginate, cellulose, etc.) that are important for biofilm formation and pathogenicity. This article will focus on the latter, also termed EPS, which are secreted to the surrounding environment, and therefore can be efficiently harvested from cell-free culture supernatant in a continuous and cost-effective manufacturing process. At present four general mechanisms are known for the production of these carbohydrate polymers in bacteria: (i) the so called Wzx/Wzy-dependent pathway; (ii) the ATP-binding cassette (ABC) transporter-dependent pathway; (iii) the synthase-dependent pathway and (iv) the extracellular synthesis by use of a single sucrase protein. The precursor molecules, which are necessary for the stepwise elongation of the polymer strands, are realized by various enzymatic transformations inside the cell, and follow in principle the same concept of producing activated sugars/sugar acids in the first three cases of different biosynthesis pathways. For the extracellular production, the polymer strand is elongated by direct addition of monosaccharides obtained by cleavage of di- or trisaccharides.

In the Wzx/Wzy dependent pathway individual repeating units, which are linked to an undecaprenol diphosphate anchor (C55) at the inner membrane, are assembled by several glycosyltransferases (GT’s) and translocated across the cytoplasmic membrane by a Wzx protein (so called flippase). In a next step their polymerization occurs at the periplasmic space by the Wzy protein before they will be exported to the cell surface (Cuthbertson et al., 2009; Morona et al., 2009; Islam and Lam, 2014). Transport of the polymerized repeat units from the periplasm to the cell surface has been shown to be dependent upon additional protein(s) assigned to the polysaccharide co-polymerase (PCP) and the outer membrane polysaccharide export (OPX; formerly OMA) families (Cuthbertson et al., 2009; Morona et al., 2009). All polysaccharides assembled by the Wzx/Wzy pathway have a highly diverse sugar pattern (up to four or five types of sugar within their chemical structure are common) and are therefore classified as heteropolymers (e.g., xanthan). All strains using this pathway carry the genes for the flippase (Wzx) and the polymerase (Wzy) within their extracellular polysaccharide operons.

**FIGURE 1:**
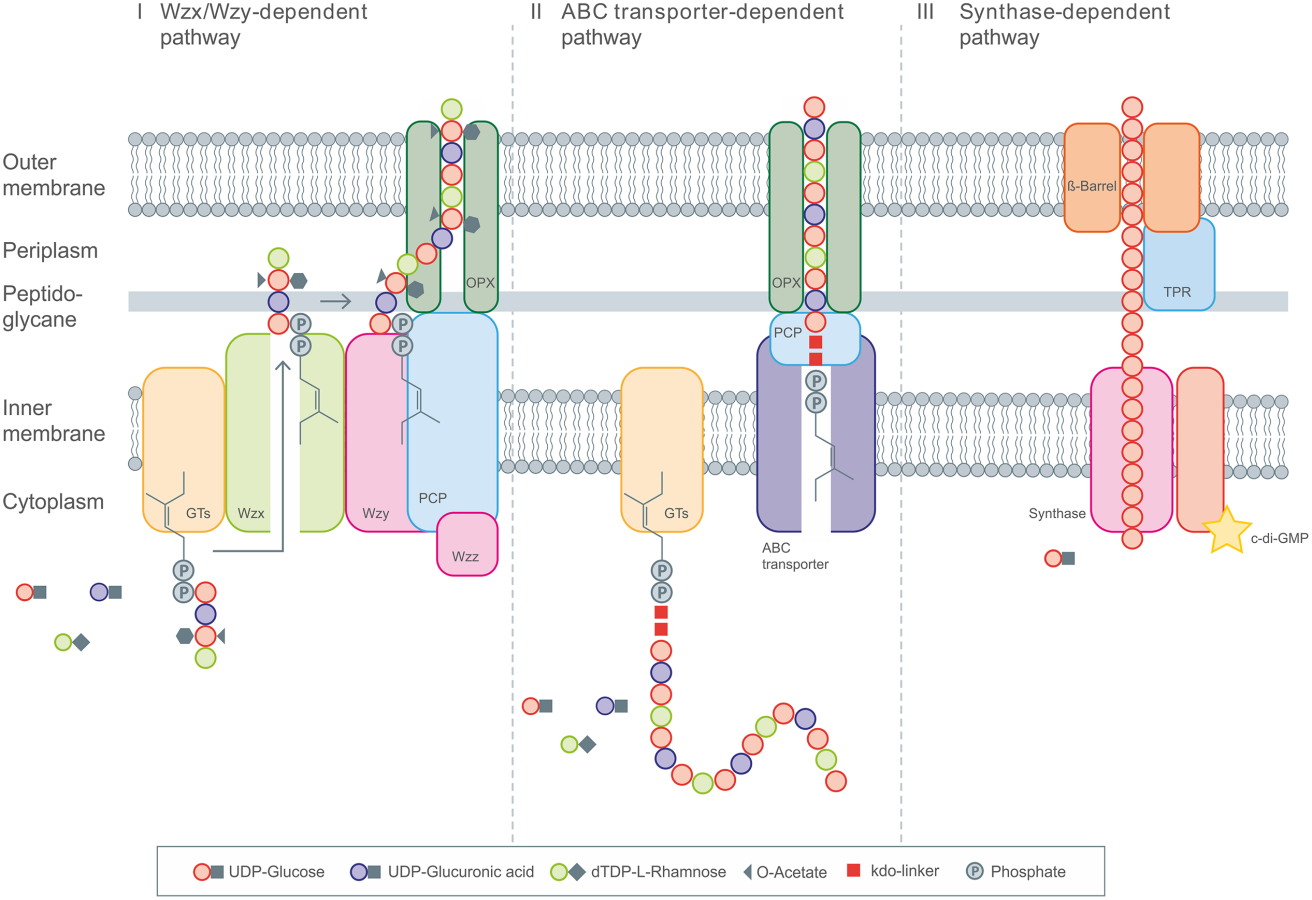
**General overview of the three different intracellular EPS biosynthesis pathways.** (I) Display the Wzx/Wzy dependent pathway with the repeating unit assembled by various Glycosyltransferases (GT’s) at the C55-lipid linker and the following translocation toward the periplasm by Wzx flippase. Polymerization occurs via Wzy polymerase and the polysaccharide co-polymerase protein (PCP). The PCP and OPX proteins realize the transport across the membranes. (II) The ABC transporter dependent pathway assembles the polysaccharide chain anchored on the poly-kdo-linker, which is then transported across the membranes and cell wall under involvement of PCP and OPX proteins. (III) The synthase dependent pathway realizes the polymerization and transport by the synthase complex, spanning the complete cell envelope, including the tetratricopeptide repeat (TPR) proteins.

The second pathway of bacterial exopolysaccharide biosynthesis is the ABC transporter dependent pathway which is mainly present in capsular polysaccharide (CPS) biosynthesis (Whitney and Howell, 2013). These polysaccharides do not really represent EPS, since they are still linked to the cell surface. Like the Wzx/Wzy dependent EPS, the CPS synthesized via the ABC-transporter dependent pathway are assembled by the action of GT’s at the cytoplasmic face of the inner membrane, resulting in homopolymers when only a single GT-containing operon is involved, or in heteropolymers when multiple GT’s are used for the assembly process (Whitney and Howell, 2013). The export across the inner membrane and translocation to the cell surface, however, is different as it is realized by a tripartite eﬄux pump like complex. The complex is composed of ABC-transporters spanning the inner membrane, and periplasmatic proteins of the PCP and OPX family (Cuthbertson et al., 2009; Morona et al., 2009). These proteins are closely related to OPX and PCP proteins involved in secretion process of the Wzx/Wzy pathway (**Figure 1**). CPSs produced via this pathway all carry a conserved glycolipid at the reducing terminus composed of phosphatidylglycerol and a poly-2-keto-3-deoxyoctulosonic acid (Kdo) linker. This represents one of the main differences of the Wzx/Wzy and the ABC dependent pathways. Just recently novel insights into the early steps in CPS biosynthesis were provided by new discoveries of the structure of this conserved lipid terminus (Willis and Whitfield, 2013; Willis et al., 2013).

The third pathway is the synthase dependent pathway, which secretes complete polymer strands across the membranes and the cell wall, and is independent of a flippase for translocating repeat units. The polymerization as well as the translocation process is performed by a single synthase protein, which in some cases (alginate, cellulose) is a subunit of an envelope-spanning multiprotein complex (Rehm, 2010). Synthase dependent pathways are often utilized for the assembly of homopolymers requiring only one type of sugar precursor. This is observed in curdlan biosynthesis for example, here only β-(1-3)-linked glucose is found in the polymer. Another example of a strict homopolymer is bacterial cellulose, consisting only of β-(1-4)-linked glucose units. In the case of alginates, the preliminary polymer is synthesized as polymannuronic acid, which is processed by different epimerases and further modifying enzymes to glucuronic/mannuronic acid block-polymers, which can differ in the ratio and sequence of G/M building blocks (Rehm and Valla, 1997). The biosynthesis of hyaluronic acid (HA) is catalyzed by a single enzyme (hyaluron synthase), which performs both steps, polymerization and secretion. Assembly of the polymeric disaccharide is realized by use of the two different precursors; glucuronic acid and GlcNAc (Chong et al., 2005). Therefore, HA synthesis differs from the other synthase dependent pathways, but at the same time shows a high degree of similarities at protein level.

Most of the enzymatic steps for exopolysaccharide precursor biosynthesis appear inside the cell while polymerization/secretion is localized in the cell envelope. But there also exist some examples of extracellular synthesized polysaccharides, such as, e.g., dextran or levan. The biosynthesis of these occurs via GT’s, which are secreted and covalently linked to the cell surface (**Table 1**).

**Table 1:** Overview of the most relevant bacterial exopolysaccharides concerning monomer composition, substituent decoration, applications, and biosynthesis pathway routes.

EPS	Components	Substituents	Applications	Biosynthesis pathway	Reference
Alginate	GulA, ManA	Ace	Food, feed, medicine, research	Synthase dependent	Rehm and Valla (1997), Rehm (2010)
Cellulose	Glc		Food, medicine, acoustics	Synthase dependent	Ross et al. (1987), Rehm (2010)
Colanic acid	Glc, Fuc, GlcA, Gal	Ace, Pyr	N.a.	Wzx/Wzy dependent	Goebel (1963), Stevenson et al. (1996), Rehm (2010)
Curdlan	Glc		Food, cosmetics, medicine, construction chemistry	Synthase dependent	Stasinopoulos et al. (1999), Rehm (2010)
Dextran	Glc		Medicine, chromatography	Extracellular, dextransucrase	Dols et al. (1998), Rehm (2010)
Diutan	Glc, Rha, GlcA,	Ace	Construction chemistry,	Wzx/Wzy dependent	Plank (2005), Pollock (2005)
Gellan	Glc, Rha, GlcA	Ace, Gly	Construction chemistry, food, feed	Wzx/Wzy dependent	Pollock (1993), Plank (2005)
Hyaluronic acid	GlcA, GlcNAc		Medicine, cosmetics	Synthase dependent	Thonard et al. (1964), Dougherty and van de Rijn (1994), Rehm (2010)
Levan	Fru, Glc		Food (prebiotic), feed, medicines, cosmetics, industry, glue	Extracellular, Levansucrase	Ceska (1971), Combie (2003), Srikanth et al. (2015)
Succinoglycan	Glc, Gal	Ace, Pyr, Suc	Oil industry, cosmetics	Wzx/Wzy dependent	Glucksmann et al. (1993b), Becker et al. (1995a)
Welan	Glc, Rha, GlcA, Man	Ace	Construction chemistry,	Wzx/Wzy dependent	Plank (2005), Pollock (2005)
Xanthan	Glc, Man, GluA	Ace, Pyr	Food, feed, technical applications, oil drilling	Wzx/Wzy dependent	Ielpi et al. (1981), Vorholter et al. (2008), Rehm (2010)

Colanic acid represents an EPS with no commercial application, but is of high interest due to pathogenicity studies. Glc, glucose; Rha, rhamnose; Fuc, fucose; Fru, fructose; Gal, galactose; Man, mannose; GlcA, glucuronic acid; ManA, mannuronic acid; GulA, guluronic acid; GalA, galacturonic acid; GlcNAc, *N*-acetyl-glucosamine; Pyr, pyruvate; Ace, acetate; Gly, glycerate; Suc, succinate, N.a., not announced.

**FIGURE 2:**
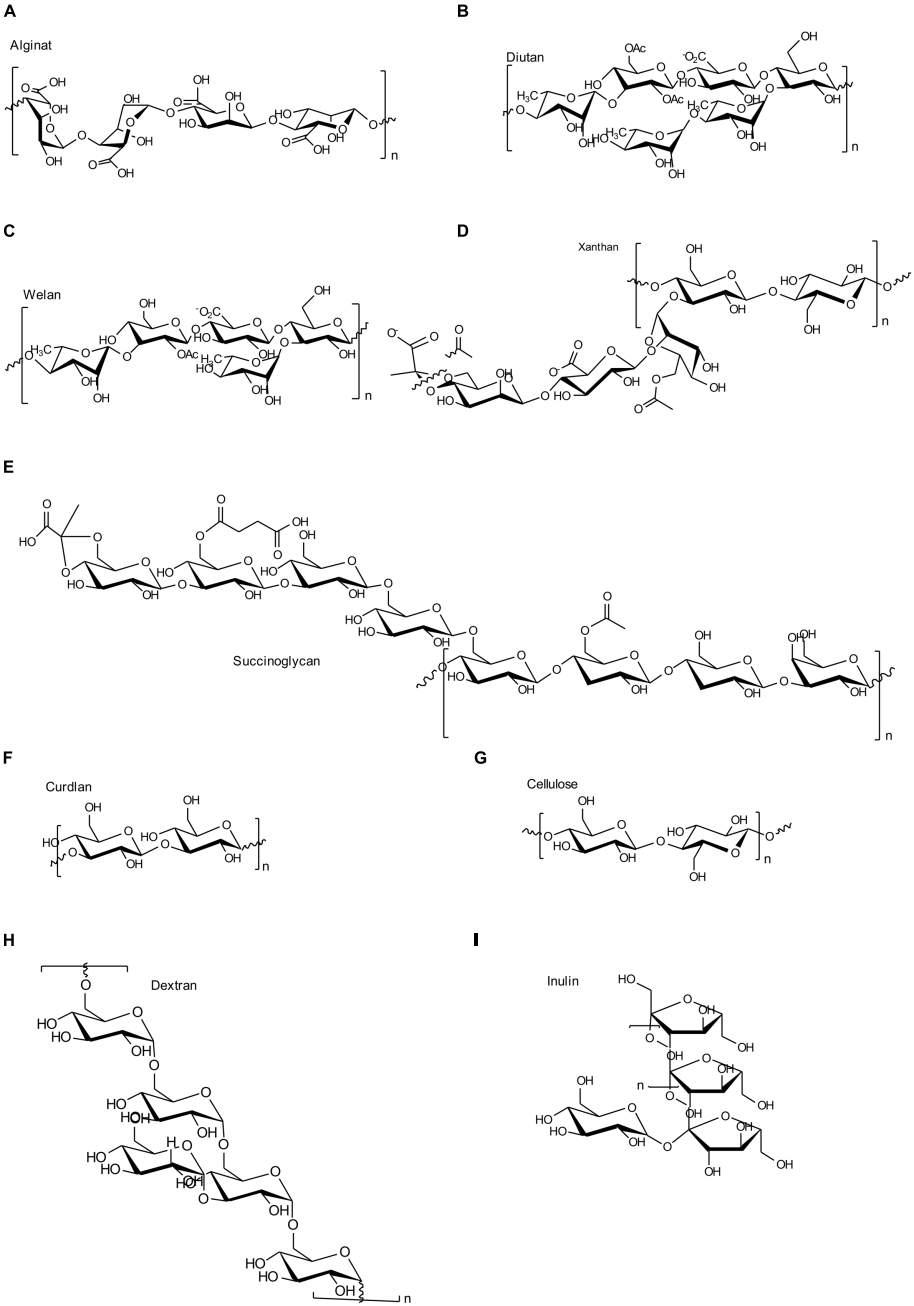
**Chemical structures of the most important EPS as described in this manuscript. (A)** Alginate; **(B)** Diutan; **(C)** Welan; **(D)** Xanthan; **(E)** Succinoglycan; **(F)** Curdlan; **(G)** Cellulose; **(H)** Dextran; **(I)** Inulin like fructan.

**FIGURE 3:**
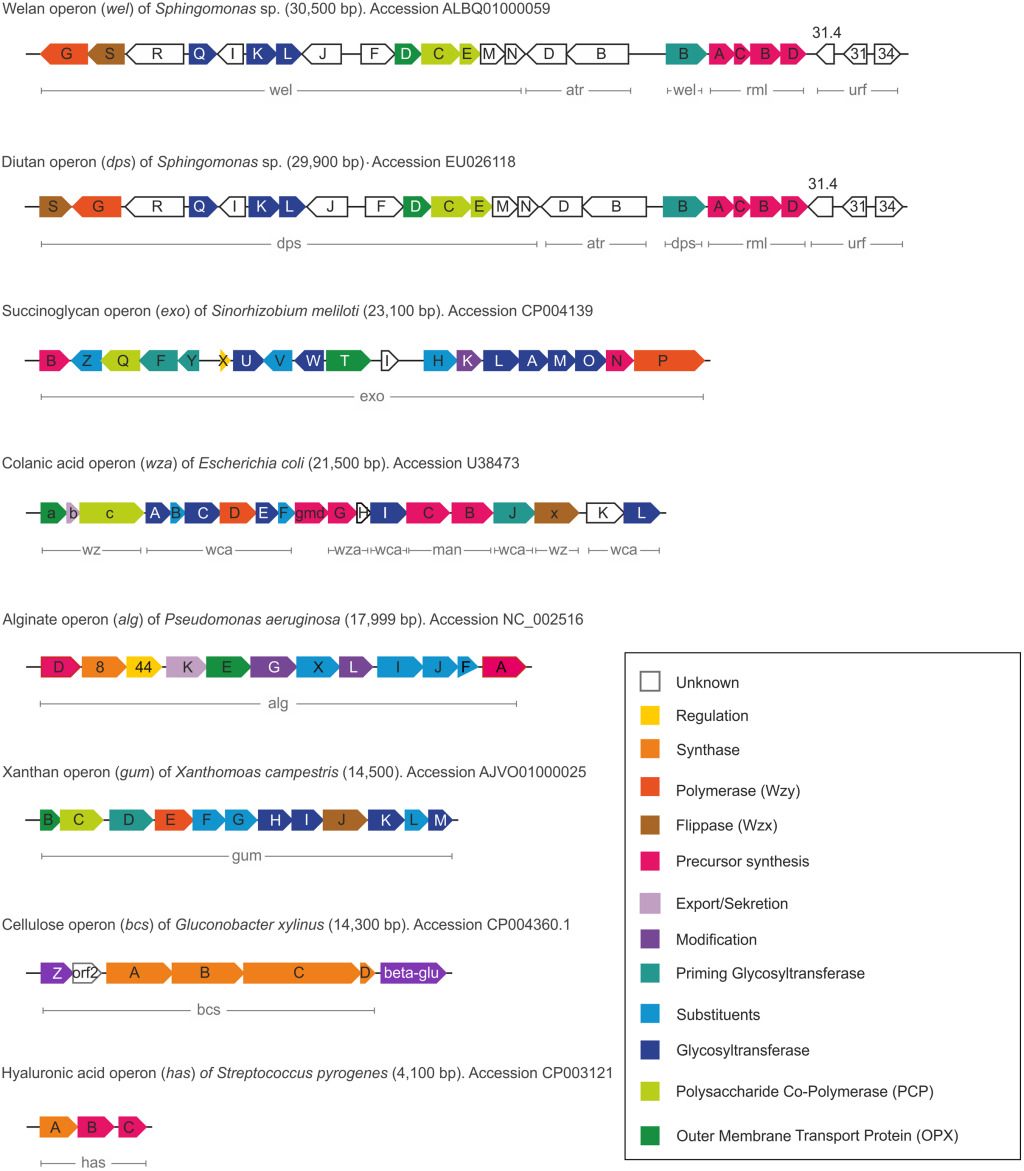
**Comparison of the different gene clusters including functions of the various encoded proteins**.

In alignment with the various EPS biosynthesis pathways, the chemical structure and material properties of the final polymers are quite variable (**Figure 2**). The genes involved in the different biosynthesis pathways encode various types of GT’s, polymerizing and branching enzymes, as well as enzymes responsible for addition of substituents or modifications of sugar moieties. Not all steps in the various pathways are currently understood, and sometimes the differences between the pathways become less defined. The genes encoding these enzymes can be found in most of the EPS producing microbes clustered within the genome or on large plasmids (Finan et al., 1986; Rehm, 2010). This condensed appearance of several GT’s and polymerizing as well as secreting enzymes (one to more than 23) facilitates the identification of EPS operons, even if only partially sequenced draft genomes are available (**Figure 3**). Even though many gene clusters responsible for EPS biosynthesis have been known for several years, the function and mode of action of most of the genes and proteins is not completely clarified. An overview of the most relevant, commercial available EPS, including the biosynthesis pathway they are produced by is given in **Table 1**.

Since the ABC transporter dependent pathway is mainly involved in the biosynthesis of (CPSs, only a schematic outline of its synthesis will be presented here, and the interested reader is referred to several outstanding reviews in this field (Whitfield, 2006; Cuthbertson et al., 2010; Willis and Whitfield, 2013).

## General Strategies for Engineering of Bacterial Polysaccharides

Bacterial polysaccharides have diverse unique properties for food applications and are used as viscosifiers, stabilizers, emulsifiers, or gelling agents. Due to these valuable properties several studies were performed to genetically engineer the producing organisms in order to generate novel polysaccharide variants and to improve production. Putative targets for engineering are the molecular weight, composition and sequence of co-monomers as well as addition of substituents. Bacterial polysaccharides by their diversity inherently exhibit a tremendous design space toward the production of new valuable materials.

Within the last years intensive research focused on providing insight into the mechanisms underlying bacterial exopolysaccharide biosynthesis pathways. High through-put genome sequencing, functional genomics, protein structure analysis and new bioinformatics tools aid toward identifying new EPS biosynthesis pathways and to understand the principles of EPS formation.

Depending on the purpose, engineering strategies can be subdivided into different categories. One goal of EPS production engineering is an increased volumetric productivity to cost-effectively produce the various EPS. These studies were mostly aiming at increasing the pool of sugar nucleotides (i.e., EPS precursors) to enhance the carbon flux toward the final polymer. In particular genes of precursor biosynthesis were overexpressed. This strategy was demonstrated to be successful for some EPS producers, but failed in some cases (Thorne et al., 2000; Videira et al., 2000; Huang et al., 2013; Schatschneider et al., 2013; Wu et al., 2014). Additionally, in some cases the overexpression of genes involved in the EPS assembly (e.g., GTs, Wzx, Wzy) resulted in enhanced yields and precursor conversion rates while in other cases it had a negative effect presumably due to distorting the multidomain protein complex involved in polymerization and secretion (van Kranenburg et al., 1999a). These approaches included overexpression of single genes as well as whole gene clusters (Pollock et al., 1996; Harding et al., 2011; Jones, 2012). Additionally the targeted engineering of regulatory proteins could increase productivity by increasing transcription of the operons, which encode the EPS biosynthesis proteins. Furthermore, the disruption of pathways competing for precursors used for EPS formation did also increase the productivity (Pena et al., 2002; Galindo et al., 2007). Single gene knock-outs were also described to enhance yield as well as to alter the chemical structure of the EPS (Nunez et al., 2009; Gaytan et al., 2012). Unfortunately, the titer of bacterial polysaccharides is limited in the production because the highly viscous polysaccharides have a massive negative influence on mass transfer (Seviour et al., 2011). However, the strategy to enhance productivity based on genetic engineering might be interesting for EPS with reduced viscosifying properties, for example due to lower molecular weight. The optimization of manufacturing process parameters might be more promising than engineering EPS biosynthesis for many established industrial EPS producers. The highest titers of highly viscous EPS such as xanthan are around 30–50 g/L (Sieber et al., 2006; Hublik, 2012) and represent the current maximum amount, which is manageable by existing bioprocess units.

Another strategy of engineering EPS biosynthesis is aiming at tailor-made variants with desirable material properties for medical and industrial applications. Here the aim is to alter the molecular structure and therefore the behavior and material characteristics of the final polymer. For example these modifications can be based on deleting substituents or monomeric sugars from the side chain. On the other hand new or more substituents might be attached to change the ratio of decoration. Most efforts were done in engineering the degree of acetylation and pyruvylation of various polymers, in order to control their rheological behavior (Hassler and Doherty, 1990; Donati and Paoletti, 2009). Additionally an altered degree of pyruvylation results in varied charge density of the polysaccharide (Skjåk-Bræk et al., 1989). Targeted modification of the molecular weight via overexpression or mutation of genes involved in the polymerization/degradation process (e.g., synthase, Wzy, PCP/lyases, glucosidases) represents another possibility to adjust rheology of the final product and was reported for some EPS (Rehm, 2010; Diaz-Barrera et al., 2012; Galván et al., 2013).

The early engineering approaches of xanthan biosynthesis as performed by Hassler and Doherty (1990) gave interesting insights in general structure-function relationships. The strongest influence on rheology was observed by altering the substituent decoration. The degree of acetylation and pyruvylation has opposite effects on viscosity. A high degree of pyruvylation resulted in higher viscosity, whereas more acetyl groups decreased viscosity of the resulting EPS. This finding is a general rule for polysaccharides and can be used in tailoring the viscosity of EPS. The degree of acetylation can be adjusted by *in vivo* as well as *in vitro* approaches or even process parameters applied during the production process (Skjåk-Bræk et al., 1989; Pena et al., 2006; Donati and Paoletti, 2009; Diaz-Barrera et al., 2010; Gaytan et al., 2012; Rehm, 2015). Further engineering approaches with respect to the production of xanthan variants included the targeted engineering of the length of the side chain. These approaches are of great interest, since they might be transferred to other EPS variants. A truncated tetramer xanthan version, obtained by deletion of the terminal mannose via inactivation of the GT GumI results in a much lower viscosity. The further removal of the glucuronic acid from the side chain by inactivation of GumK (a GT) resulted in enhanced viscosity compared to the wild type when measured as a function of concentration (Hassler and Doherty, 1990). The influence of acetylation on viscosity of the truncated versions showed an irregular result. The acetylated polytetramer version showed decreased viscosity as observed for the wild type xanthan. The acetylation of the polytrimer (only one mannose as side chain) resulted in similar viscosity as the non-acetylated version. Pyruvylation of the outer mannose also blocks acetylation of this sugar, therefore enhancing the viscosity. In the wild type xanthan gum acetylation of the inner or the outer mannose showed similar viscosities, which indicated that the extend of acetylation affected viscosity, but the position within the polymer is less critical. Whether the change in viscosity results from the substituents itself, or from conformational changes of the polymers remains elusive up to now. Several studies describe the occurrence of conformational changes by side chain pyruvylation and acetylation (Morris et al., 1977; Lecourtier et al., 1986; Muller et al., 1986; Liu et al., 1987). Just recently, the molecular weight of xanthan was synthetically adjusted by controlling the expression level of the Wzy polymerase GumE (Galván et al., 2013). For alginate a similar effect was observed by an overexpression of alginate polymerase *alg8/alg44* in *Azotobacter vinelandii* resulting in a high molecular weight alginate variant (Diaz-Barrera et al., 2012).

Relatively little information is available on EPS with varying monomer composition. The transfer of complete gene clusters toward alternative host strains was reported to result in altered compositions of the repeat units (Pollock et al., 1997; van Kranenburg et al., 1999a). This effect might result from the different enzymatic equipment of the host strains for nucleotide precursor synthesis (Stingele et al., 1999). These heterologous expression strategies were mostly combined with lowered production levels of the foreign polymers (Stingele et al., 1999). Complementation experiments of single GTs and further proteins involved in the biosynthesis pathway were performed (van Kranenburg et al., 1999a,b). These results showed a relatively broad specificity of the Wzx and Wzy proteins in regard of the chemical structure of the repeating units in several strains, indicating a high potential for modifying these and still using the same secretion and polymerization machinery.

## Exopolysaccharides Produced Via the Wzx/Wzy-Dependent Pathway

### Xanthan – A Highly Diverse Heteropolysaccharide with Long Side Chain

Xanthan gum as produced by *Xanthomonas campestris* consists of a cellulose like backbone [β-(1-4)-linked glucose] and a side chain made of two mannose units and one glucuronic acid (Jansson et al., 1975; **Figure 2D**). Xanthan is produced by the two precursor’s glucose- and fructose 6-phosphate, the key intermediates of the central carbohydrate metabolism. At the moment five different genomes of *X. campestris* pv. *campestris* are available (Schatschneider et al., 2013). Comparative genomics of three of these identified a common core genome of about 3,800 genes, with a diverse amount (∼500) of unique genes, but simultaneously highly conserved xanthan operon and *xanAB* genes (precursor synthesis; Vorholter et al., 2008). Just recently the draft genome of *X. campestris* NRRL B-1459 (ATCC 13951) was published which might further enhance the insights in conserved xanthan biosynthesis pathway (Wibberg et al., 2015). *X. campestris* is capable of utilizing a vast amount of carbohydrates (Vorholter et al., 2008) and several transcriptomic and genome wide analytical approaches were performed for *X. campestris* strains (Chung et al., 2007; Serrania et al., 2008; Vorholter et al., 2008; Zhou et al., 2011; Li et al., 2014). Just recently there was published a large scale *in silico* based metabolomics network, verified by experimental data (Schatschneider et al., 2013). This model gave further insight into stimulated growth and xanthan production in complete accordance with the experimental data for xanthan as well as biomass production. This verified model represents the first one focusing on microbial polysaccharide biosynthesis and might dramatically enhance the knowledge for generalized enhanced product titers.

The biosynthesis pathway as encoded by the *gum* cluster comprises 13 genes involved in assembly of the repeat unit, polymerization, translocation as well as decoration with substituents. The bifunctional genes providing the nucleotide precursors (*xanAB*) are not located within the gum-cluster. In detail, the assembly of the pentasaccharide repeating unit starts with the transfers of the first glucose unit toward the phosphorylated lipid linker (C55) anchored to the inner membrane via the priming GT GumD. In a next step, the cytosolic GT GumM attaches the second glucose unit by a β-(1-4)-bond to the first glucose. Catalyzed by GumH the first mannose unit is linked by an α-(1-3)-glyosidic bond, followed by the cytosolic glycosyltransferase activity of GumK which adds a β-(1-2)-linked glucuronic acid. Finally the repeating unit is completed by action of GumI, attaching the terminal mannose via a β-(1-4)-bond. In general most of the GTs involved in biosynthesis of EPS following the Wzx/Wzy–pathway appear to be monofunctional and the same applies specifically also for the xanthan biosynthesis (Breton et al., 2006). The genes encoding GumF, GumG, and GumL are known to be involved in acetylation and pyruvylation of the repeating units of xanthan. The GumL protein is known to incorporate pyruvyl residues to the external β-mannose, while the acetyl residues are incorporated into the internal α-mannose by GumF, and into the external β-mannose by GumG (Becker et al., 1998). Whether the decoration with substituents occurs before the spatial reorientation toward the periplasm or within the periplasm is not elucidated up to now. In some cases there was observed a decreased activity of the GT’s when acetylated precursors were used as in the case of GumK (Barreras et al., 2004). These findings might indicate that at least a final repeat unit is necessary for decoration, but the last proof of spatial action of GumFGL still remains speculative.

The translocation process of the complete repeating unit is realized by the flippase GumJ (Wzx-protein) at which the repeat unit is still linked to the C55 anchor, which might play an important role in the targeted transport of the repeating unit (Islam and Lam, 2014).

The general topology of Wzx proteins shows several transmembrane helices (TMHs), 10 in the case of GumJ (Vorholter et al., 2008). Data of tertiary structure, which will give further insights into the mechanism and functionality of these highly complex membrane proteins, is still missing and only a low amount of homology models is available (He et al., 2010; Islam and Lam, 2014). Polymerization of the translocated repeating units occurs via the action of GumE, a membrane protein, showing 12 TMHs and a large periplasmatic loop as described for other Wzy-proteins (Whitfield, 2006; Vorholter et al., 2008; Islam et al., 2010; Marczak et al., 2013). The exact mechanism as well as substrate specificity of GumE and most other Wzy-proteins remain elusive up to now.

A putative adaption mechanism toward the length, as well as acceptance of repeating units with modified side chains was observed for some Wzy-proteins, characterizing them to be well suited for acceptance of tailored repeating units as obtained by genetic engineering (Nyman et al., 1979; Reeves et al., 2013; Islam and Lam, 2014).

The PCP proteins as present in the Wzx/Wzy pathway are assumed to be responsible for chain length control of the final polymer (Cuthbertson et al., 2009) and much more information is available compared to Wzx and Wzy proteins, even on structural level (Islam and Lam, 2014; Schmid and Sieber, 2015). GumC as inner membrane protein belongs to the PCP-2a sub-family. These proteins are distinguished by their common topology, which consist of a large periplasmatic domain, flanked by two transmembrane fragments. The PCP-2a sub-family normally shows an additional C-terminal cytoplasmic kinase domain, which is not the case for GumC topology (Cuthbertson et al., 2009; Galván et al., 2013). This domain is normally autophosphorylated at several tyrosine residues and seems to be essential for assembly of high molecular weight EPS, rendering GumC to be somehow different (Cuthbertson et al., 2009; Bianco et al., 2014). For further reinforcement of this finding, no kinase partner has been identified in the *X. campestris* genome (Cuthbertson et al., 2009). The general characteristics of the different gum genes as identified in *X. campestris* are given in **Table 2**.

**Table 2:** General characteristics of the several *gum* genes as present in the *xanthan gum*-cluster from *Xanthomonas campestris*.

Gene	Length (aa)	Localization	Protein family	Mechanism	Additional information	Reference
GumM	263	Cytosol	CAZY 26	Transferase, inverting	PFAM WecB-GT family	Becker et al. (1998), Vorholter et al. (2008)
GumH	380	Cytosol	CAZY 4	Transferase, retaining	PFAM GT family 1	Becker et al. (1998), Vorholter et al. (2008)
GumK	400	Membrane	CAZY 70	Transferase, inverting	Membrane associated	Barreras et al. (2004), Vorholter et al. (2008)
GumI	349	Membrane	CAZY unclassified	Transferase, putative retaining	GT-B, Monotopic	Becker et al. (1998), Vorholter et al. (2008)
GumJ	492	Membrane	Wzx	Flippase	PFAM PS-biosynthesis protein family (10 TMHs)	Becker et al. (1998), Vorholter et al. (2008)
GumE	428	Membrane	Wzy	Polymerase	10–12 TMHs predicted	Becker et al. (1998), Vorholter et al. (2008)
GumC	479	Membrane	PCP2a	Export	Oligomeric, no glycosylation	Vorholter et al. (2008), Galván et al. (2013)
GumB	232	Membrane	OPX-C	Export	Tetramer, PES domain	Jacobs et al. (2012), Galván et al. (2013)
GumD	484	Membrane	Undecaprenyl-Glc-GT	Transferase	Priming GT	Ielpi et al. (1981, 1993)
GumL	264	Membrane?	Pyruvyltransferase	Transferase		Harding et al. (1987), Becker et al. (1998), Vorholter et al. (2008)
GumF	364	Membrane	Acetyltransferase	Transferase	PFAM family 3, 9 TMHs	Vorholter et al. (2008)
GumG	351	Membrane	Acetyltransferase	Transferase	PFAM family 3, 9 TMHs	Vorholter et al. (2008)

Length in amino acids is given for the proteins as identified in *Xanthomonas campestris* JX.

Just recently the crystal structure of the soluble form of GumB was published, revealing its structure to be a tetramer of ∼100 kDa (Jacobs et al., 2012; Bianco et al., 2014). GumB represents the corresponding OPX proteins as necessary for the final stage of polymer secretion (Vorholter et al., 2008). GumB is an OPX protein containing the polysaccharide export sequence (PES) motif which is characteristic for OPX proteins and can be categorized to the OPX-C family as defined by Cuthbertson et al. (2009). Interestingly there are no transmembrane regions identified by *in silico* prediction, but GumB is located in membrane fractions as identified by subcellular location experiments (Galván et al., 2013). The OPX and PCP protein (GumB and GumC) comprise a molecular scaffold that spans the cell envelope (Cuthbertson et al., 2009). Early engineering approaches already revealed the absolutely necessity of *gumB* and *gumC* in xanthan biosynthesis. No xanthan production was observed when *gumB* or *gumC* were inactivated, but assemblage of the repeat unit was still realized (Katzen et al., 1998). Co-overexpression of *gumB* and *gumC* results in higher molecular weight xanthan as well as higher viscosity, therefore indicating direct interaction of both proteins (Galván et al., 2013). Even if more and more information of the interplay of GumB and GumC is available, there would still be the need for further experiments to elucidate the interaction and functionality of this trans- periplasmic/membrane spanning complex.

### Sphingan – A Family of Similar but Different Heteropolysaccharides

**FIGURE 4:**
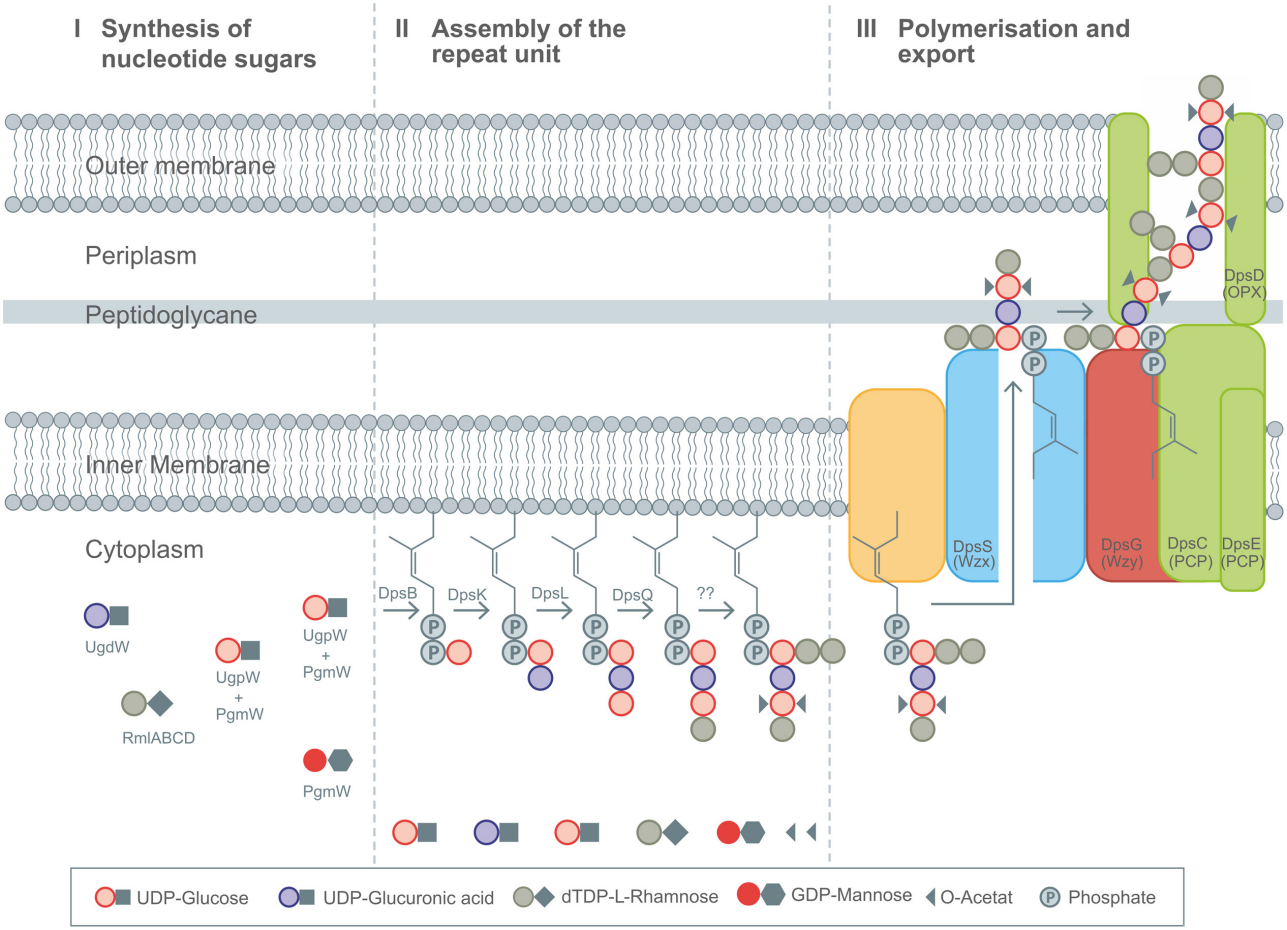
**Detailed description of the Wzx/Wzy-pathway as present in diutan biosynthesis by *Sphingomonas sp.* ATCC 53159.** (I) Synthesis of nucleotide sugars in the cytoplasm. (II) Stepwise assembly of the repeating unit at the membrane bound C55 linker including decoration with substituents by action DpsBKL and DpsQ. The proteins involved in branching and transfer of acetyl groups remain elusive up to now. (III) Polymerization and translocation process across the membranes as guided by Wzx (DpsS), Wzy (DpsG), PCP (DpsC and DpsE), and OPX (DpsD) proteins. Figure modified according to Fialho et al. (2008), Schmid et al., (2014a,b), and Schmid and Sieber (2015).

Different heteropolysaccharides with closely related chemical structures, but strongly differing material properties belongs to the family of sphingans as produced by several *Sphingomonas* and *Pseudomonas* strains, (Pollock, 2005). The backbone of most sphingans is composed of Rha-Glc-GlcA-Glc (Pollock, 2005) with small variation in the sugar composition of the backbone (Rha or Man) as well as the side chains, when existent (Jansson et al., 1983, 1986; O’Neill et al., 1983; Jansson and Widmalm, 1994). EPS included in the sphingan family are gellan, welan, diutan, rhamsan, S-7 and S-88 (Pollock, 2005). The differences in the chemical structures are encoded in differently composed gene operons, as just recently reviewed (Schmid et al., 2014b). The genes involved in the synthesis of the rhamnose precursor (*rmlABCD*) are placed on the highly conserved gene operon; the genes necessary for the other nucleotide sugar precursors are randomly distributed within the genome (Harding et al., 2004; Wang et al., 2012a). The assembly of the repeat unit of the different sphingans follows a strict procedure, encoded in the corresponding sphingan operons. In the case of the three sphingans, gellan, welan and diutan, the genes involved in the biosynthesis are named according to the corresponding polymer, *gel* for gellan, *wel* for welan and *dsp* for diutan, respectively. To facilitate the general description of the biosynthesis of this highly similar EPS the introduction of gene nomenclature *spn*, for genes involved in sphingan biosynthesis was suggested and will be used here (Schmid et al., 2014b). The assembly of the identical backbone of the repeat unit for gellan, welan and diutan occurs by the transfer of glucose toward the C55-anchor, via the priming glycosyltransferase SpnB. In a next step, the glucuronic acid is linked toward the priming glucose by a β-(1-4)-bond catalyzed by SpnK. As a third glycosyltransferase involved in the assembly process, SpnL transfers the second glucose to the nascent repeat unit and finally SpnQ transfers the rhamnose unit by linking it via an α-(1-3)-bond (**Figure 4**).

The next steps are different in gellan, welan and diutan. Gellan represents the unbranched version of sphingans, which only shows substituent decorations of glycerol and acetyl at the second of the two glucose units of its backbone.

Welan is described to carry only one acetyl group as substituent, and a side branch of α-(1-3)-linked rhamnose or mannose in the ratio of 2:1 as present at the first glucose of the repeat unit (Stankowski and Zeller, 1992; Jansson and Widmalm, 1994). Diutan has a dimeric L-rhamnose side chain attached to the first glucose residue of the growing repeating unit (Chowdhury et al., 1987) and two acetyl groups are attached per repeating unit to the C2 and C6 positions of the second glucose in the repeating unit (Diltz and Zeller, 2001; **Figures 2C,D**). The genes involved in incorporation of the side chains for welan and diutan have not been exactly functionally assigned at present. But the genes *urf31.4*, *urf31,* and *urf34*, which are labeled as “unknown reading frames” are assumed to be involved (Coleman et al., 2008). These findings are in accordance with the different amount of *urf* genes present in the *spn* operons encoding the differently branched sphingan variants (Harding et al., 2003, 2004; Coleman et al., 2008; Schmid et al., 2014b). Up to now only one acetyltransferase, outside the *spn* operon was verified to be involved in acetylation of gellan (Harding et al., 2003).

Whether the addition of the side chain sugars and non-sugar substituents occurs as final step of repeat unit assembly or at the nascent repeat unit remains speculative up to now, but as observed for xanthan, it can be assumed that already decorated repeat unit intermediates might reduce the activity of the GT’s involved in assembly of the repeat unit (Barreras et al., 2004). The next steps of sphingan biosynthesis follow the same order for all variants and include activity of the Wzx-protein flippase (SpnS), the Wzy-polymerase (SpnG) as well as the PCPs, which are thought to be SpnC and SpnE and seem to be involved in chain length regulation, having the typical kinase domains in their sequences (Moreira et al., 2004). The complete mechanism of their function is still speculative. Secretion of the finally polymerized sphingans occurs via OPX protein SpnD and the two PCP proteins which comprise a molecular scaffold spanning the cell envelope.

### Succinoglycan – A Heteropolysaccharide with Large Repeating Unit

Succinoglycan (SG) is an acidic EPS produced by several *Rhizobium*, *Agrobacterium*, *Alcaligenes,* and *Pseudomonas* strains (Harada and Yoshimura, 1964; Zevenhuizen, 1997). The model organism for SG production is *Rhizobium meliloti* RM 1201. SG is a branched heteropolysaccharide consisting of an oligosaccharide repeat unit with several substituent decorations, such as succinate, pyruvate and acetate. The monomers included in the repeat unit are β-linked glucose and galactose in the ratio of 7:1 (**Figure 2E**). Pyruvate is present in stoichiometric ratio, whilst succinate and acetate decoration depends on strain origin and culture conditions (Stredansky, 2005). The pyruvate residues are linked at the positions O4 and O6 of the terminal glucose residue of the side chain. SG plays an essential role in plant symbiosis (Leigh and Walker, 1994) and next to its biological importance shows industrially attractive properties (Sutherland, 1990; Stredansky and Conti, 1999). Biosynthesis of SG uses a set of 19 genes, which are labeled as *exo* genes, and two additionally *exs* genes which are also involved in SG biosynthesis (Glucksmann et al., 1993b; Becker et al., 2002). *ExoC*, *exoB* and *exoN* are involved in the biosynthesis of the precursors UDP-galactose and UDP-glucose encoding the corresponding phosphoglucomutase, UDP-glucose-4-epimerase and UDP-pyrophosphorylase, respectively (Uttaro et al., 1990; Buendia et al., 1991; Stredansky, 2005). ExoY represents the priming GT, which initiates the synthesis of the repeating unit by transferring the galactosyl residue to the lipid carrier having a high similarity on nucleotide level to the priming GT GumD in xanthan biosynthesis. Interestingly ExoY needs the gene product of *exoF* to successfully transfer the galactose toward the lipid linker (**Figure 3**). The genes *exoA*, *exoL*, *exoM*, *exoO*, *exoU,* and *exoW* code for the GTs, subsequently elongating the octasaccharide repeating unit by addition of a glucose unit (Glucksmann et al., 1993a; Reuber and Walker, 1993). ExoP (PCP-2a), ExoQ (Wzy), and ExoT (OPX) represent the necessary translocation end polymerization machinery, including chain length control of SG (Becker et al., 1995b, 2002; Niemeyer and Becker, 2001). The ExoP protein has the typical periplasmic domain flanked by two transmembrane regions and an additional cytoplasmic domain. It shows autophosphorylating protein tyrosine kinase activity and is involved in molecular weight distribution of SG (Niemeyer and Becker, 2001) The transmembrane proteins ExoZ, and ExoH decorate the repeating unit with acetyl and succinyl substituents. ExoV protein is a ketalase transferring a pyruvyl group to the terminal glucose (Leigh et al., 1987; Glucksmann et al., 1993b). Interestingly, SG producing strains encode genomic information for extracellular endoglycanases (ExoK and ExsH), to reduce the high molecular weight of the product (Becker et al., 1993; York and Walker, 1997; Jones et al., 2007).

The majority of the 21 genes involved in SG biosynthesis are clustered on a megaplasmid (Glucksmann et al., 1993b; Becker et al., 1995a) and only *exoC* is located on the chromosome (Glucksmann et al., 1993a). The *exs* genes can be found adjusted to the *exo* genes upstream of the *exoB* gene. Two of the *exs* (*exsA*, *exsB*) genes were identified to be involved in SG biosynthesis, *R. melioti* strains carrying mutated *exsA* (high similarity to ABC-transporter) variants showed an altered ratio of high molecular SG to low molecular SG, indicating involvement of *exsA*, without further knowledge of the detailed function. *ExsB* gene product was shown to have a negative influence on SG biosynthesis, resulting in lowered product titers (Becker et al., 1995a). The phenomenon of plasmid based EPS operons is widespread, especially in the field of *Lactobacilli* (Kranenburg et al., 1997). Another gene cluster encoding the second EPS of *Rhizobium* (galactoglucan) is localized on the same megaplasmid, but more than 200 kb away (Charles and Finan, 1991; Becker et al., 1997). This EPS also consists of galactose and glucose, but in the ration of 1:1 (Chandrasekaran et al., 1994).

This phenomenon of the genetic equipment for the production of more than one EPS is also very widespread amongst microbes (Christensen et al., 1985; Sutherland, 2001; Wozniak et al., 2003b; Laue et al., 2008) and complicates the defined analysis of sugar moieties making up the polymer (Rühmann et al., 2015).

### Colanic Acid

Colanic acid (CA) also is known as the M antigen and is described to be an EPS (Goebel, 1963). CA is mainly found in *Enterobacteria* and is made from a repeat unit of glucose, fucose, galactose, and glucuronic acid. Decorations of acetyl and pyruvate are present as substituents in non-stoichiometric amount (Grant et al., 1969; Garegg et al., 1971). CA biosynthesis has been linked to a cluster of 19 genes mainly named following the general nomenclature for polysaccharide biosynthesis genes as suggested by Reeves et al. (1996). The genes for synthesis of the fucose nucleoside sugar precursors are placed within the CA gene cluster (Stevenson et al., 1996; Stout, 1996). The genes *manB* and *manC* are directly involved in the biosynthesis mechanism of GDP-mannose, which is converted via a three-step pathway toward GDP-fucose (Stevenson et al., 1996; Andrianopoulos et al., 1998). These three steps are catalyzed by GDP-mannose dehydratase (GMD) followed by an epimerase and reductase reaction as catalyzed by the bifunctional *wcaG* gene, which encodes the fucose-synthase (Andrianopoulos et al., 1998; Albermann et al., 2000). The genes for the synthesis of the other nucleotide precursors can be found dispersed in the genome. The stepwise assembly of the repeat unit is initiated via the action of WcaJ, which will transfer the first glucose unit toward the C55 lipid carrier (Johnson and Wilson, 1977; Patel et al., 2012). The next sugar monomers will be transferred by the action of WcaA, WcaC, WcaE, WcaI, and WcaL. The order of synthesizing steps is not completely clarified and mainly is based on sequence similarities and not on biochemical experiments (Stevenson et al., 1996). Due to its location within the fucose synthesizing genes, WcaI might be involved in transfer of fucose units (Stevenson et al., 1996). The structural similarity as found for WcaL suggest involvement in transfer of galactose or glucuronic acid (Stevenson et al., 1996).

For WcaB and WcaF a high similarity with the family of acetyltransferases is observed, but no precise role of WcaB or WcaF in acetylation process or explanation for presence of two acetyltrasnferases is given up to now (**Figure 3**). The Wzx protein was identified within the CA gene cluster by its typical transmembrane segments and the large periplasmic loop. WcaD is predicted to span the inner membrane with nine transmembrane segments, and to polymerize the repeat units of CA (Stevenson et al., 1996), therefore representing the Wzy polymerase. The OPX protein involved in the secretion process in concert with the PCP proteins is encoded by *wza* and can be categorized as OPX group A protein, which can functionally replace its homolog in K30 biosynthesis pathway (Reid and Whitfield, 2005; Cuthbertson et al., 2009). Wzc forms the typical contiguous molecular scaffold that spans the cell envelope together with Wza and belongs to the PCP-2a family. The Wzb protein represents the protein tyrosine phosphatase, which controls the phosphorylation state of Wzc, the corresponding tyrosine kinase. The detailed regulatory interactions between Wzb and Wzc were recently characterized for the first time (Temel et al., 2013). Several characterization and mutation experiments were performed for the K30 analog of Wza and Wzc, giving further insights into mechanism and structure (Whitfield, 2006; Cuthbertson et al., 2009; Willis and Whitfield, 2013; Willis et al., 2013).

## Exopolysaccharides Produced Via the Various Synthase-Dependent Pathways

### Homopolysaccharides

#### Curdlan, A Bacterial β-(1-3)-Glucan

Curdlan is a water insoluble β-(1-3)-glucan (glucose homopolymer) without any substituents, produced by, e.g., *Agrobacterium* (**Figure 2F**). Four genes are involved in curdlan biosynthesis (*crdA*, *crdS*, *scrdR,* and *crdB*). The curdlan synthase (CrdS), is the key enzyme of curdlan biosynthesis, showing a high similarity to cellulose synthases (Stasinopoulos et al., 1999). The lack of experimental characterization of curdlan synthase makes it difficult to determine its mechanism of biosynthesis and secretion (Whitney and Howell, 2013). Many genomic as well as transcriptomic and proteomic information is available for curdlan producing *Agrobacterium* strains (McIntosh et al., 2005; Zheng et al., 2007; Jin et al., 2008; Wu et al., 2008; Ruffing and Chen, 2010, 2012; Ruffing et al., 2011; Jin and Lee, 2014). Results as obtained by that approaches displayed metabolic structures and pathway distributions indicating that energy efficiency, rather than substrate availability is the major constraint for improving curdlan yield (Zheng et al., 2007). Curdlan displays an EPS of high industrial interest and product titers of around 70 g/L were reported (Wu et al., 2008). The biosynthesis via the CrdS is believed to occur by the repetitive addition of glucosyl residues from the sugar nucleotide donor UDP-glucose to form polymeric β-(1-3)-glucan chains (Hrmova et al., 2010). The CrdS is confined within cell membranes and belongs to GT2 family of glycosyltransferases, predicted to adopt a GT-A fold and using an inverting reaction mechanism that is mediated through a single displacement reaction via a glycosyl-enzyme intermediate (Coutinho et al., 2003). First insights into the putative structure and mechanism were obtained and might enhance the understanding of curdlan synthases in the future (Hrmova et al., 2010). Additionally a cell-free protein synthesis was recently realized for the curdlan synthase in nanodiscs and was followed by X-ray scattering to obtain further structural information (Periasamy et al., 2013).

#### Cellulose, A Bacterial β-(1-4)-Glucan

Cellulose is a major component of several bacterial biofilms and has been increasingly considered as biomaterial for medical applications. The first description of cellulose synthesizing (*celS*) genes from *Acetobacter xylinum* (*acsABCD*) was given in Wong et al. (1990). These encoded proteins showed very low sequence identity to the corresponding plant homologues (<30%) of the cellulose synthases. The bacterial cytoplasmic membrane cellulose synthase (Bcs) proteins also belong to the GT2 family and are composed of three subunits (BcsA, BcsB, and BcsC; Ross et al., 1991). In some bacterial species BcsA and BcsB can be found fused as a single polypeptide (Umeda et al., 1999; Kawano et al., 2002). Cellulose biosynthesis occurs by polymerization of UDP-glucose nucleotide sugar precursors (Umeda et al., 1999). *BcsA* encodes the catalytic subunit of cellulose synthase and binds UDP-glucose to guarantee supply of monomers for polymerization. BcsB represents the regulatory subunit of the synthase complex, whereas the functional role of BcsC and BcsD are not functionally assigned yet. BcsC is proposed to function as pore formation protein to enable cellulose secretion, whereas BcsD seems to be involved in control of crystallization process of cellulose nanofibrils (Lee et al., 2014). Additionally, the genes *bcsZ* encoding for an endo-β-(1-4)-glucanase and the *orf2* encoding for the “cellulose completing protein” are placed up-stream of the cellulose synthase operon (Kawano et al., 2002). Both genes are essential for cellulose biosynthesis (Kawano et al., 2002). The function of *orf2* has not been determined up to now, but is essential in cellulose synthesis (Standal et al., 1994). Located downstream from the cellulose synthase operon is a gene encoding a β-glycosidase, which hydrolyzes glucose units consisting of more than three monomers and has an essential role in cellulose production (Kawano et al., 2002). The cellulose synthesizing operon as recently identified in *Gluconacetobacter xylinus* E25 (Kubiak et al., 2014) is given in **Figure 3**. The typical GT-A family catalytic fold of cellulose synthase has been verified by crystallization experiments (Cantarel et al., 2009) and is composed of two juxtaposed β/α/β-folds which form a β-sheet surrounded by α-helices (Lairson et al., 2008). Morgan et al. (2013) the crystal structure and the reaction mechanisms of the cellulose synthase CelS (A and B subunits) from *Rhodobacter sphaeroides* were solved. It was shown that the large central domain of BcsA contains a single UDP-glucose binding site, and that cellulose synthesis occurs within the cytosolic domain of BcsA. BcsB is located in the periplasm and might assist in guidance of the cellulose chain during the secretion process (Slabaugh et al., 2014). Several functional motifs as well as the function of different conserved amino acids were elucidated (Sethaphong et al., 2013). The GT-A fold comprised the motifs DDG and DCD, which coordinate the UDP as well as the essential divalent cation. An internal helix, which is very close to these conserved motifs, interacts with the cellulose acceptor substrate. These findings supported the hypothesis of cellulose elongation at the non-reducing end, by a single active side. The characteristic cellulose structure (**Figure 2G**) can be explained for the first time by the steric environment presented by the preceding glucose and the β-(1-4)-linkage, thus reversing the direction of terminal glucose rotation with every additional glucose monomer (Slabaugh et al., 2014).

### Heteropolysaccharides

#### Alginate, A Gel-Forming Exopolysaccharide Produced Via an Envelope-Embedded Multiprotein Complex

Alginates are EPS made of variable amounts of (1-4)-linked β-D-mannuronic acid and its C5-epimer α-L-guluronic acid (**Figure 2A**). These comonomers are arranged in blocks of continuous mannuronic acid residues (M-blocks), guluronic acid residues (G-blocks), or as alternating residues (MG-blocks; Rehm and Valla, 1997; Gutsche et al., 2006). Alginates are synthesized by brown seaweeds and by bacteria belonging to the genus *Pseudomonas* and *Azotobacter* (Rehm, 2009).

The arrangement and sequence of comonomer residues and in particular the formation of G-blocks were similar in algal and *A. vinelandii* alginates. However, alginates derived from *pseudomonads* do not contain G-blocks (Skjak-Braek et al., 1986). Based on these structural differences the various alginates adopt different material properties which align with their biological role such as, e.g., the formation of a tough cyste wall (a dormant stage) in *A. vinelandii* or the viscous biofilm matrix material in regard to *pseudomonads*.

**FIGURE 5:**
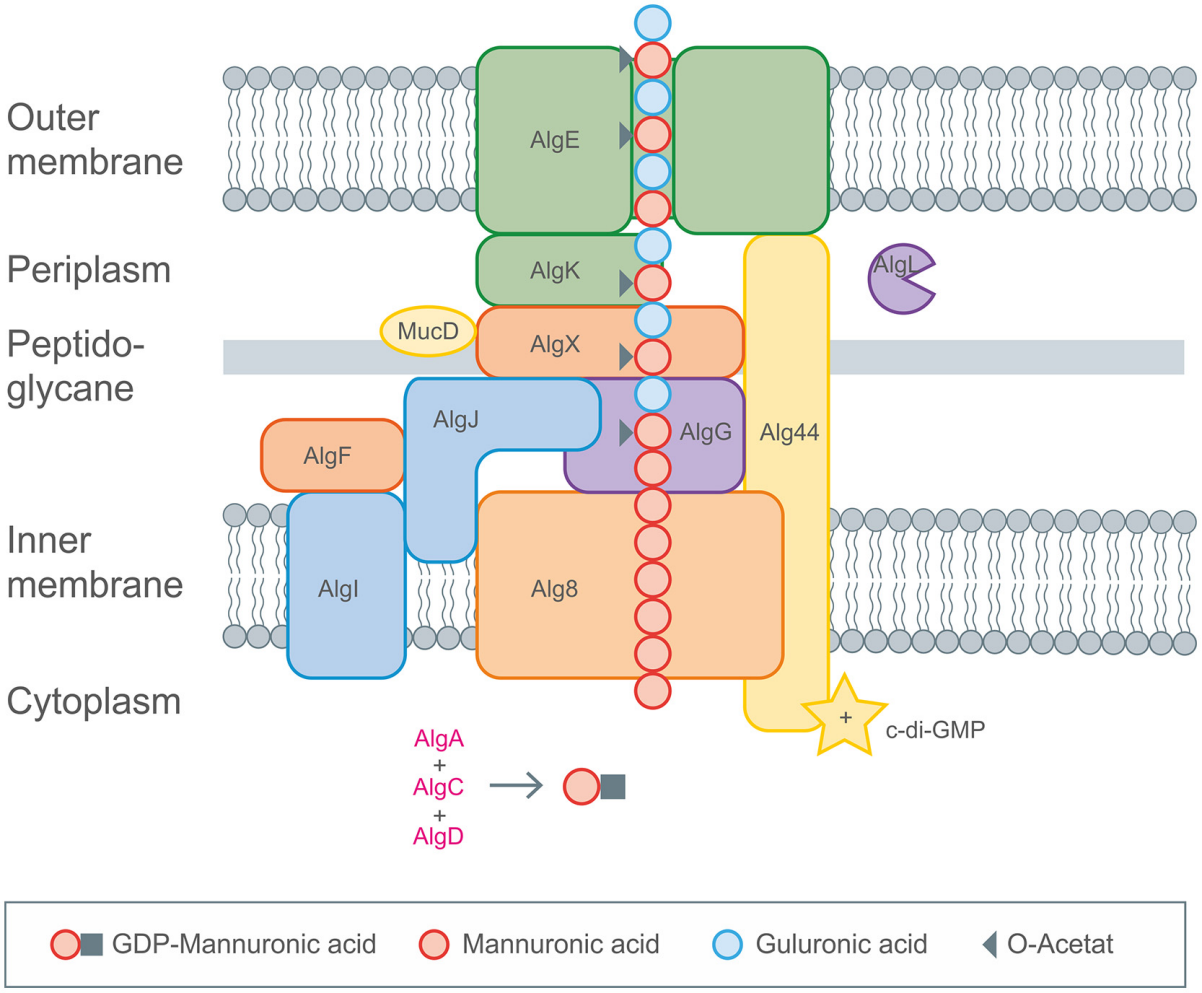
**Detailed description of alginate biosynthesis as performed by *Pseudomonas aeruginosa*.** The activated mannuronic acid precursors obtained by cytoplasmic enzyme activities are polymerized by the membrane anchored GT Alg8. The transport across the membranes occurs by the inner membrane spanning protein complexes (Alg8, Alg44), the periplasm spanning (AlgX, AlgK, AlgG, AlgL) and the outer membrane (AlgE) proteins. Decoration with acetyl groups and epimerization toward glucuronic acid occurs via AlgIJX and AlgG, respectively. Figure modified according to Hay et al. (2013).

The intracellular biosynthesis steps of the activated precursor GDP-mannuronic acid are well known (Hay et al., 2013, 2014). In contrast the molecular mechanisms underlying polymerisation and modification are less understood. Recent studies on secretion of alginate illuminated the underlying concepts based on the involvement of the secretion pore AlgE in the outer membrane of *Pseudomonas aeruginosa* (Rehm et al., 1994; Hay et al., 2010a; Whitney et al., 2011; Rehman and Rehm, 2013). Polymerisation and secretion are linked via an envelope-spanning multiprotein complex composed of at least six subunits (Alg8, Alg44, AlgG, AlgX, AlgK, AlgE) as was shown by protein–protein interaction and mutual stability studies (Gutsche et al., 2006; Hay et al., 2012, 2013; Rehman et al., 2013). A schematic overview of alginate biosynthesis pathway is given in **Figure 5**.

GDP-mannuronic acid biosynthesis starts from the central metabolite acetyl-CoA which is converted to oxaloacetate via the citric acid cycle. Oxaloacetate enters gluconeogenesis leading to fructose-6-phosphate which is then converted by alginate-specific enzymes into the activated alginate precursor GDP-mannuronic acid (Rehm and Valla, 1997). GDP-mannuronic acid is polymerized to alginate presumably by membrane-anchored Alg8, a GT, which is a subunit of the multiprotein complex (Remminghorst and Rehm, 2006b; Remminghorst et al., 2009). Only recently experimental evidence for the multiprotein complex spanning the cytoplasmic membrane (Alg8, Alg44), the periplasm (AlgX, AlgK, AlgG, AlgL) and the outer membrane (AlgE) was obtained (Rehman and Rehm, 2013). A scaffold of periplasmic proteins has been proposed to guide the nascent alginate chain through the periplasm for secretion by the alginate-specific channel protein AlgE in the outer membrane (Gutsche et al., 2006; Rehman and Rehm, 2013; Rehman et al., 2013). AlgE is linked to the periplasmic scaffold via an interaction with the lipoprotein AlgK which was found to interact with AlgX which links the periplasmic scaffold to the polymerase subunits via an interaction with Alg44. Alg44 has been described as co-polymerase which also binds c-di-GMP required for activation of alginate polymerisation (Remminghorst and Rehm, 2006a; Merighi et al., 2007). Recently, the membrane-anchored sensor protein MucR was found to be required to specifically activate alginate formation by generating a localized c-di-GMP pool likely in proximity to Alg44. However, regulation of alginate biosynthesis is highly complex and recruits a network and cascades of integrated regulatory processes as has been recently reviewed in Hay et al. (2014). It involves two-component signal transduction systems FimS/AlgR and KinB/AlgB, transcriptional regulation through various DNA-binding proteins, σ/anti-σ factors, posttranscriptional regulation through the Gac/Rsm sRNA system, posttranscriptional regulation through a natural antisense transcript (MucD-AS) that promotes alginate production by blocking the translation of *mucD* mRNA in addition to the previously mentioned c-di-GMP mediated activation. Directly following the model for xanthan biosynthesis there was published a genome-scale model for mapping the global effects of MucA on transcriptional level in *P. fluorescens*, giving great insights into correlation of alginate biosynthesis and biomass production (Borgos et al., 2013).

Bacterial alginate production could be increased by overexpressing biosynthesis genes and by inactivation of negative regulators (Hay et al., 2010b). Controlling expression of alginate modifying enzymes (lyases, epimerases, acetyltransferases) as well as their use *in vitro* enables the production of alginates with tailored molecular weight, M/G arrangement and acetylation degree, i.e., alginates exhibiting a range of desirable material properties can be obtained (Rehm, 2010). Future research will target to engineer stable and improved bacterial production strains toward biotechnological production of a wide range of defined alginates suitable for high value medical applications (Rehm, 2005). The unique material properties of alginates has been already harnessed for variety of industrial applications such as, e.g., stabilizing, thickening, and gelling agent in food production, or to immobilize cells in pharmaceutical and biotechnology industries (Paul et al., 1986). Commercial alginates are currently exclusively produced from brown seaweeds.

#### Hyaluronic Acid, A Heteropolysaccharide Following the Synthase Dependent Pathway

Hyaluronic acid or hyaluronan is a linear, extremely hydrophilic polymer of alternating β-D-glucuronic acid and β-D-*N*-acetyl-glucosamine residues linked via β-(1-4) and β-(1-3)-glycosidic bonds (Atkins and Sheehan, 1971; Toole, 2004; Weigel and DeAngelis, 2007). HA can be produced by vertebrates and prokaryotes and finds a wide range of applications especially in medicine and cosmetics due to its biocompatibility, high water retention capacity and excellent viscous behavior. The HA operon is composed of three genes, but for assembly of the polymer only the cytosolic membrane embedded HA synthase (HasA) is the key player (DeAngelis and Weigel, 1994; Chong et al., 2005; Liu et al., 2011). The genes *hasC* and *hasB* encode for UDP-glucose pyrophosphorylase, catalyzing the synthesis of UDP-glucose from UTP and glucose-1-phosphate and the UDP-glucose dehydrogenase activity, catalyzing the oxidation toward UDP-glucuronic acid, respectively.

UDP-N-acetyl-glucosamine results from phosphorylation reactions encoded by genes of the global cell metabolism (Chong et al., 2005). The bacterial HasA proteins (Class I) are GTs integrated in the membrane polymerizing the precursor molecules by adding new moieties to the reducing end of the polysaccharide chain (Weigel and DeAngelis, 2007; Badel et al., 2011). As cellulose and curdlan synthases they contain a core of four TMHs connected to at least one intracellular loop, which contains the consensus sequence of processive GT’s (Saxena et al., 1995; Heldermon et al., 2001). Different hypothesis for the HA synthase reaction mechanism exist, such as combined glycosyltransferase and translocase activity (Tlapak-Simmons et al., 1999; Weigel and DeAngelis, 2007; Thomas and Brown, 2010) or inclusion of an HA secreting ABC transporter (Ouskova et al., 2004; Schulz et al., 2007). Just recently it was shown by *in vitro* analysis based on proteoliposomes that the synthesis and translocation process spatially aligned (Hubbard et al., 2012). In combination with the latest results as obtained for cellulose and curdlan synthases, the mechanism of HA synthesis might soon be solved.

The first commercial bacterial HA was produced via *Streptococcus zooepidemicus,* but due the production of streptolysin (exotoxin), causing β-hemolysis, as observed during cultivation of streptococci strains, recombinant HA production was of high priority even in the early stage of commercialization (Thonard et al., 1964; Widner et al., 2005; Chien and Lee, 2007; Liu et al., 2011). The high-price segments in which HA can be applied motivated metabolic engineering approaches to enhance and tailor HA synthesis (Chong et al., 2005), identifying a balanced availability of both precursor molecules to influence yield and molecular weight (Chen et al., 2009; Jia et al., 2013).

## Extracellularly Synthesized Polysaccharides

### Dextran and Derivatives – Sucrase Based Glucan-Homopolymers

The most common sucrase activity based polymer is dextran, which mainly consists of α-(1-6) linked glucose (**Figure 2H**). Polymerization is realized outside the cell by the dextransucrase the key enzyme for dextran synthesis. Dextransucrases (or more general glucansucrases) belong to the enzyme class of transglucosidases that are part of the glucosyltransferases which are classified as glycoside hydrolase family 70 (GH70; Cantarel et al., 2009). Glucansucrases (GS) catalyze the transfer of glucose from sucrose onto a growing chain of α-glycosidic linked oligo- and polysaccharides. Depending on the glucansucrase the resulting linkages can be formed to each of the free hydroxyl groups of the sugar moiety. Besides the purely α-(1-6) linked dextran there are dextrans containing a small amount of α-(1-3) or even α-(1-2) linkages, mutan with mostly α-(1-3) linkages, alternan with strictly alternating α-(1-3) and α-(1-6) linkages and reuteran consisting mainly of α-(1-4) linkages that are interspersed with α-(1-6) linkages (Dols et al., 1998; Leemhuis et al., 2013). The polymers formed from glucansucrases are branched to different degrees with possible branching points at all hydroxyl groups.

Dextransucrases are secreted and anchored to the cell wall. They have average molecular weights in the range of 110–160 kDa and are built of multiple domains (Vujičić-Žagar et al., 2010). Recently three dimensional structures of truncated GH70 proteins have become available (Vujičić-Žagar et al., 2010; Ito et al., 2011; Brison et al., 2012) and the structural models revealed surprisingly different structures than expected from sequence alignments. The catalytic core of the GSs consists of three domains, with two extra domains attached to the core domains (Leemhuis et al., 2013). Only the C-domain consists of one contiguous polypeptide and is involved in glucan production and/or glucan binding (Lis et al., 1995; Kingston et al., 2002; Kralj et al., 2004).

There has been some debate over the years on the enzymatic mechanism, especially concerning chain initiation and chain elongation. Some evidence was provided that elongation occurs at the reducing end, which led to the proposal of two nucleophilic sites being involved, where the growing chain remains covalently bound to the enzyme and is transferred from site one to the glucose moiety bound at site two and *vice versa* (Robyt et al., 2008). This mechanism would explain the high processivity of the enzyme as polymer length is inversely proportional to the number of enzymes (Robyt et al., 2008). Other studies and the recently solved crystal structures of glucansucrases, however, propose a much simpler mechanism (Leemhuis et al., 2013). Here, during elongation sucrose is hydrolyzed resembling the action of a retaining glycosyltransferase where a covalent β-glycosyl-enzyme intermediate is formed via a carboxylic acid residue of the enzyme. Within this step the high energy of the glycosidic bond of sucrose is retained. Hydrolysis of this intermediate with concomitant release of glucose is possible but not favored by the enzyme. Instead a transfer occurs of the glucose onto any suitable hydroxyl group. Accordingly elongation occurs at the non-reducing end. In this reaction model the acceptor hydroxyl group is determined by what molecule is bound to the active site and how this is held in position, which might be mediated by a carbohydrate binding module in the multidomain protein (Janecek et al., 2011). This model would also explain the branching to be a more statistic phenomenon caused by movement of the chain or by intermediate release and rebinding. The mechanism of initiation has also been debated and different monomers and oligomers have been found or proposed as primers, depending on the experimental setup. Since the presence of bound oligomers seems to promote the transfer reaction the initiation should occur on various molecules with sufficiently high concentration to bind (Seibel et al., 2006).

#### Levan and Inulin

Sucrases can also act as fructose transferases producing polyfructan. Two types of linkages have been described here, α-(2-6) and α-(2-1). Levan, which is produced by levansucrases, mainly consist of the former with occasional α-(2-1) branches. Inulin type polyfructan is obtained from inulinosucrase and shows the opposite α-(2-1) chain with α-(2-6) branches (**Figure 2I**). Levansucrases are widely distributed among Gram-positive bacteria and several plant pathogens carry more than one enzyme (Khandekar et al., 2014); inulinosucrases are only present in lactic acid bacteria (Srikanth et al., 2015). Mechanism of reaction is similar to glucansucrases. When fed with maltose as sole priming substrate fructansucrases can also produce maltosylfructosides (Diez-Municio et al., 2013).

The synthesis of dextran, levan and derivatives is directly induced in the presence of sucrose for some *Leuconostoc mesenteroides* strains (Kim and Robyt, 1994) and some strains express the genes constitutively (Schwab et al., 2007).

## Biosynthesis Regulation

Regulation of the various EPS biosynthesis pathways is complex and occurs at different levels. Some general regulation strategies such as extensive transcriptional regulation involving two-component signal transduction pathways, quorum sensing, alternative RNA polymerase σ-factors and anti-σ-factors, as well as integration host factor (IHF)-dependent and cyclic di-GMP dependent regulatory mechanisms are available and will be summarized here.

Nitrogen limitation was shown to induce EPS biosynthesis for many different EPS producing strains by use of the different components of the nitrogen signaling cascade (Ruffing and Chen, 2012). Additionally, and in general the presence of numerous c-di-GMP synthesizing diguanylate cyclase proteins which contain the conserved GGDEF motif as catalytic active side (Ausmees et al., 2001; Simm et al., 2004) play an important role in regulation of EPS-biosynthesis (Hengge, 2009; Ruffing and Chen, 2012; Liang, 2015). Transcriptomic analysis of **curdlan** biosynthesis displayed up-regulation of GGDEF protein encoding genes under nitrogen limited conditions and lowered EPS production (57%) by knocking out c-di-GMP synthases (Ruffing and Chen, 2012). Several EPS such as cellulose, alginate, and xanthan are regulated by c-di-GMP, which can serve as an allosteric regulator of cellulose and alginate synthases (Hay et al., 2014; Liang, 2015). *Agrobacterium* sp. ATCC 31749 for example encodes 31 proteins containing GGDEF domains (Ruffing and Chen, 2012). The regulation of EPS biosynthesis strongly differs amongst the bacterial species as indicated by the highly varying amount of genes encoding for c-di-GMP synthesizing and degrading proteins (EAL or HD-GYP motifs). This amount ranges from zero in *Staphylococcus aureus* to more than 80 in *Kineococcus radiotolerans*, as recently reviewed (Liang, 2015). *Xanthomonas* genomes encode around 40 of these proteins for example.

The nitrogen regulatory mechanisms are centrally controlled by conserved sets of nitrogen sensor proteins called PII and the signal is transmitted by a two component signal transduction mechanism involving NtrB and NtrC. In the cascade NtrC is phosphorylated by NtrB under nitrogen deplete conditions. Nitrogen regulatory operon (*ntrBC*) also includes *nifR* whose function is unknown. Knock-outs results in 30% reduction of curdlan production, whereas the knock-out experiments of σ-factor *rpoN* result in a 30% increased curdlan biosynthesis (Ruffing and Chen, 2012). Similar to alginate, the regulation circuits of curdlan are expected to be multifaceted. Curdlan biosynthesis is dependent on pH-value and the acidocalcisome as well as polyphosphate. Knock-out experiments of a putative acidocalcisome gene (*rrpP*, membrane bound proton translocating pyrophosphatase) resulted in a 70% decrease in curdlan production. Energy storage via polyphosphate influences curdlan biosynthesis as well as maintains the intracellular pH (Ruffing and Chen, 2012). Just recently it was shown that the helix-turn-helix transcriptional regulatory protein (*crdR*) is essential for curdlan production by operating as positive transcriptional regulator of the curdlan operon in ATCC31749. The potential binding region of *crdR* is located upstream from the *crdA* start codon (Yu et al., 2015).

Several EPS synthases/copolymerases contain so called PilZ domains, which bind c-di-GMP, as shown for example for Alg44 in alginate biosynthesis (Merighi et al., 2007; Hay et al., 2009) and for AcsA/BcsA in **cellulose** synthesis, within BcsA’s C-terminus right next to the GT-domain as well as several more structural hints for binding possibilities (Weinhouse et al., 1997; Amikam and Galperin, 2006; Ryjenkov et al., 2006; Fujiwara et al., 2013; Morgan et al., 2013). This stimulating effect is assumed to be based on an induced conformational change of BcsA that allows UDP-glucose to access the catalytic site more effective (Morgan et al., 2014).

**Alginate** biosynthesis is controlled by one of the best examined regulatory networks due to the clinical significance of *P. aeruginosa*. The regulatory network of alginate biosynthesis is highly complex and occurs on different levels (Hay et al., 2014). The master regulator of alginate biosynthesis is the alternative σ-factor AlgU, classified as an extra cytoplasmic function σ -factor, which confers resistance to several environmental stress factors affecting the cell envelope (Hay et al., 2014). AlgU promotes the expression of several genes involved in alginate biosynthesis (*algD*, *algC*), regulation (*algR*, *algB*, *algZ*) as well as its own operon (Yu et al., 1997; Firoved et al., 2002; Wozniak et al., 2003a; Muhammadi and Ahmed, 2007). The anti σ-factor MucA is embedded in the cytoplasmic membrane and directly binds to AlgU and exert its function in concert with MucB, MucC, and MucD (Boucher et al., 1996; Wood and Ohman, 2006; Cezairliyan and Sauer, 2009). The complete process of regulation of alginate biosynthesis based on envelope stress following the complex regulated intermembrane proteolysis (RIP) cascade was recently reviewed in detail (Hay et al., 2014). Next to AlgU, transcriptional regulation occurs by the activity of the sigma factors AlgQ, which blocks the general housekeeping σ-factor RpoD (σ 70), thus allowing AlgU to mediate transcription of the alginate biosynthesis operon (Pineda et al., 2004; Yin et al., 2013). In nitrogen rich conditions the alternative σ-factor RpoN (σ 54) binds to *algD* promotor and therefore suppresses alginate production (Boucher et al., 2000). The alginate biosynthesis operon expression is further regulated by several DNA-binding proteins occupying the promoter region (Kato et al., 1990; Baynham et al., 2006). Other DNA-binding proteins, such as AlgQ and AlgP are described to be positive regulators of alginate biosynthesis (Deretic et al., 1992; Delic-Attree et al., 1997; Ledgham et al., 2003; Yin et al., 2013). Another DNA-binding protein (Vfr) is more indirectly involved in regulation of alginate biosynthesis with a more speculative mechanism (Hay et al., 2014). Additionally two-component signal transduction systems are involved in alginate biosynthesis regulation as observed by the autophosphorylation mechanism of the sensor kinase proteins KinB and FimS, which activate the corresponding response regulators AlgB and AlgR respectively. The detailed mechanism remains elusive up to now, but AlgB and AlgR are known to activate expression of alginate biosynthesis genes by binding to *algD* promotor (Hay et al., 2014). Generally, the stimulation of two-component systems is induced by environmental signals, which are not clearly identified for AlgB and AlgR (Wozniak and Ohman, 1991; Leech et al., 2008). Posttranscriptional regulation is observed by non-coding small RNAs whose transcription is activated by a two-component system (GacS-GacA), known to be involved in regulation of several independent pathways including such as, e.g., oxidative stress and quorum sensing (Timmermans and Van Melderen, 2010; Ghaz-Jahanian et al., 2013). The posttranslational regulation of alginate biosynthesis occurs by the already described c-di-GMP binding to the PilZ domain of Alg44 and the assumed conformational change, which might bring the nucleotide sugar closer to the active site of the corresponding glycosyltransferase active site (Alg8) as recently shown for the BscA protein in cellulose synthesis (Morgan et al., 2014). A corresponding cytoplasmic GGDEF protein was recently identified, which might provide the necessary pool of c-di-GMP, and at the same time might be involved in c-di-GMP degradation by having an additional EAL motif (Galperin et al., 2001; Hay et al., 2009; Li et al., 2013).

For ***Xanthomonas*** strains several genome wide expression profiles were recorded under different conditions, revealing single ORFs of the gum operon to be transcribed as polycistronic mRNA, from *gumB* to *gumN*, while glucose seems to induce xanthan biosynthesis (Yoon and Cho, 2007; Serrania et al., 2008; Liu et al., 2013). in this plant pathogenic bacterium the virulence and exopolysaccharide production are closely connected and controlled by cell–cell signaling mediated by diffusible signal factors (DSFs) and an uniquely evolved two-component regulatory system (RpfC/RpFG; Barber et al., 1997). The DSF signaling is mediated by c-di-GMP and the two-component system RpfC/RpfG senses and transduces the DSF signal. RpfG contains a HD-GYP domain indicating regulation via c-di-GMP. Additionally, the global transcriptional regulator Clp mediates the DSF signaling through a hierarchical regulatory network, directly acting on xanthan production (He et al., 2007). Knowledge with respect to the extensive metabolic network might be used toward a better understanding of the regulation of xanthan biosynthesis (Schatschneider et al., 2013).

Factors inducing **CA** biosynthesis were identified to mainly rely on stress conditions for cell envelope structure (osmotic stress) and seem to be mainly controlled by the Rcs (regulation of capsule synthesis) proteins, as reviewed by Majdalani and Gottesman (2005). The complex signal transduction system comprises several proteins, whereas RcsA, RcsB, and RcsD play the major role in CA biosynthesis (Majdalani and Gottesman, 2005). RcsC is a sensor protein which directly interacts with the response regulator RcsB, the signal for RcsC response is still unknown (Arricau et al., 1998; Chen et al., 2001; Majdalani and Gottesman, 2005). A helix-turn-helix DNA binding motif of the RcsB protein is phosphorylated by RcsC when stimulated by environmental cues (Takeda et al., 2001; Clarke et al., 2002). The phosphorylated version of RcsB interacts with RcsA and forms a heterodimer by utilization of an additional helix-turn-helix DNA motif as present in RcsA (Stout et al., 1991). Both positive regulators RcsA and RcsB act as a heterodimer to activate transcription at the single promoter upstream of the CA operon (Stout, 1996; Rahn and Whitfield, 2003).

One of the main differences of CA regulation is the absence of CA production in wild-type strains grown at 37°C, whereas cultivation at lowered temperatures seems to induce CA biosynthesis (Whitfield, 2006). This temperature sensitivity was examined by RT-PCR experiments, showing that expression of the CA operon was only slightly altered by growth at 19, or 37°C. Additionally the expression profile of the Rcs machinery was only marginally altered. Navasa et al. (2011), were providing experimental evidence for the underlying mechanism.

Regulation of **succinoglycan** is occurring mainly at transcriptional and posttranscriptional level (Keller et al., 1995; Becker et al., 2002; Janczarek, 2011) and employs by several proteins involved in different regulatory mechanisms. The *exoS* encoded membrane sensor together with the gene product of *chvI* (response regulator) constitute a two-component regulatory system which controls the expression of the *exo* genes. ExoR as a negative regulator directly acting on transcription and translation levels of most of the *exo* genes with exception of *exoB* (Reuber and Walker, 1993). Another posttranscriptional regulatory effect such as lowered EPS yields was observed by overexpression of *exoX* and increased EPS yields by overexpression of *exoY*, respectively without influencing any of the genes involved in succinoglycan biosynthesis (Reed et al., 1991; Leigh and Walker, 1994). The *mucR* encoded regulatory protein that plays a key role in control of biosynthesis EPS of *Rhizobium* (Keller et al., 1995). MucR causes overexpression of *exoK*, *exoY,* and *exoF* genes, resulting in high production levels. Expression can be induced by ammonia, phosphate and sulfate present in the cultivation media. Increased succinoglycan production was recently obtained by overexpression of the priming GT *exoY* (Jones, 2012).

Negative regulation of the single promotor controlling **HA** synthesis was shown to involve the two-component regulatory system CovR/S (Dougherty and van de Rijn, 1994; Heath et al., 1999). This system consist of the CovS sensor kinase and the response regulator CovR, which binds to the AT-rich elements of the *has* promotor (Bernish and van de Rijn, 1999; Miller et al., 2001; Federle and Scott, 2002). Just recently novel elements including two additional promotors, one initiating transcription of a small RNA, and an additional intrinsic transcriptional terminator were identified (Falaleeva et al., 2014). Negative regulation occurs via CovR.

Production of the extracellular **sucrases** can be induced by sucrose, which directly induces reversible phosphorylation of the sacY anti-terminator as observed in many *Bacillus* species (Idelson and Amster-Choder, 1998; Biedendieck et al., 2007). Additionally high salt concentrations induce sucrose expression by activating the DegSU-two component system (Biedendieck et al., 2007). For other genera the involvement of the negative regulators RcsA and RcsB is described to reduce the amount of levansucrase (Bereswill and Geider, 1997). Differences in Gram-negative and Gram-positive bacteria were observed, as well as dependency of expression in the stationary phase (Abhishek et al., 2009). For some levansucrases a temperature dependent expression is described which occurs at around 18°C.

## Bioengineering Strategies Toward Tailor-Made Exopolysaccharides

In addition to the already existing techniques for realization of tailor-made variants of microbial polysaccharides (exchange/overexpression of single genes or complete operons), approaches implementing domain shuﬄing of GTs revealed to be of high potential to enhance the portfolio of EPS variants (Hancock et al., 2006). The increasing insights into fundamental mechanisms and functions of genes and proteins involved in the different EPS producing biosynthesis pathways in combination with the growing portfolio of techniques unraveling metabolic networks have the potential to be applied for the production of tailor-made polysaccharides. Additionally, identification of novel EPS encoding gene clusters by next generation sequencing approaches will enhance our understanding of EPS synthesis pathway variation and modification. Combining bioinformatics, with high throughput data obtained by systems biology approaches as well as structural information of proteins and EPS will enable the implementation of synthetic biology approaches for tailoring microbial EPS. Synthetic biology approaches such as utilization of regulatory bio bricks might enable the targeted induction of EPS biosynthesis, aiming to uncouple it from natural regulatory networks. In the case of heteropolysaccharides, the complete biosynthesis machinery might be used to design tailor-made polysaccharides. Growing insights into mechanism and structures of the various GT’s as involved in assembly of the repeat units will enable the targeted shuﬄing of GT’s and therefore might enable the design of artificial repeat units in the future. The insights in Wzx and Wzy topology and mechanism might open up the opportunity for incorporation of desired sugars or sugar derivatives resulting in modified EPS structures with hitherto unknown material properties (Marolda et al., 2006; Hong et al., 2012; Wang et al., 2012b; Hong and Reeves, 2014; Islam and Lam, 2014; Rehm, 2015). In combination with the enhanced understanding of EPS biosynthesis regulation as obtained by systems and synthetic biology approaches, a novel era of bacterial EPS might be reached. Completely synthetic gene clusters could be obtained by application of domain swapping approaches, exchange of elements between different gene clusters as well as introduction of additional elements.

Alternatively protein engineering can be applied to EPS modifying enzymes for *in vivo* as well as *in vitro* modification of EPS. The latter strategy was successfully applied to alginate and succinoglycan, both having in common the secretion of enzymes for EPS modification. Utilization of these secreted enzymes might allow a tight control of the material properties. Epimerases of *A. vinelandii* have been employed to modify alginate exhibiting a range of material properties (Martinsen et al., 1989; Morch et al., 2007; Reese et al., 2015). Such variants can be of particular interest for specific applications because of their specific material properties. Future engineering strategies of alginate variants will include the design of block-copolymers to obtain tailored properties. This might include other monomers in addition to guluronic acid and mannuronic acid in order to broaden the range of applications. For homopolymers such as, e.g., curdlan and cellulose, targeted modification of the synthases involved in the biosynthesis process might enable the modification of the molecular weight as well as selectivity of glyosidic bonds within the backbone structure. As seen by the latest results from insights into the cellulose synthase machinery, the strict order of β-(1-4)-glyosidic linkages might be modified by altering the substrate binding pocket of the synthase, which is responsible for the orientation of the UDP-glucose. These structural insights might also give further information for related GT2 proteins, such as involved in the synthesis of hyaluronan, alginate, and chitin (Weigel and DeAngelis, 2007; Hubbard et al., 2012; Hay et al., 2014).

Only recently an innovative bi-enzymatic process was reported for the production of short chain fructooligosaccharides and oligolevans from sucrose. This system was based on an immobilized levansucrase and an endo-inulase, resulting in a highly efficient synthesis system with a yield of more than 65% and a productivity of 96 g/L/h (Tian et al., 2014). The utilization and combination of several carbohydrate modifying enzymes create the potential for industrial production of different low molecular weight oligo- or polysaccharides with applications as food additives (prebiotics) or in medicine and for industry.

## Conflict of Interest Statement

The authors declare that the research was conducted in the absence of any commercial or financial relationships that could be construed as a potential conflict of interest.
